# Using healthcare-affiliated apps to recruit typically underrepresented populations in a pregnancy cohort study in West Virginia^☆^

**DOI:** 10.1016/j.conctc.2025.101535

**Published:** 2025-08-07

**Authors:** Katrina L. Wilhite, Elly Marshall, Carly Williamson Rogers, Kara M. Whitaker, Lindsay Morris-Neuberger, Bethany Barone Gibbs

**Affiliations:** a Department of Epidemiology and Biostatistics, West Virginia University, Morgantown, WV, United States of America; b Department of Exercise Physiology, University of Pittsburgh, Pittsburgh, PA, United States of America; c Department of Health and Human Physiology, University of Iowa, Iowa City, IA, United States of America; d Department of Communication Studies, West Virginia University, Morgantown, WV, United States of America

**Keywords:** Recruitment, Rural, Socioeconomic status, Pregnancy

## Abstract

**Background:**

Individuals residing in rural areas and those with low socioeconomic status are typically underrepresented in clinical research. Strategies to recruit more representative populations should be explored. This study aimed to compare screening and consenting proportions in a West Virginia-based pregnancy cohort study of individuals sent a recruitment message via the MyChart patient portal, overall and stratified by rurality and insurance status (proxy for socioeconomic status).

**Methods:**

Between October 2022 and April 2023, 1024 recruitment messages were sent via MyChart, a healthcare-affiliated portal. Screening and consenting proportions were calculated overall and stratified by rurality and insurance status. Logistic regression was used to compare participation proportions.

**Results:**

The recruiting method yielded 2.8 % consent. Screening and consenting proportions were similar across stratified groups.

**Conclusions:**

Healthcare-affiliated portals may be a useful strategy to recruit typically underrepresented populations in pregnancy-related research.

## 1 Introduction

Reducing health inequities and promoting physical health and well-being for everyone are key public health goals [1,2]. To achieve these goals, researchers must include typically underrepresented groups (e.g., the financially and socially disadvantaged, people living in rural communities, and women) who tend to experience poorer health outcomes in clinical research [[3], [4], [5]]. Historically, recruiting participants from typically underrepresented groups, particularly racial and ethnic minorities, for interventions has been challenging for researchers studying a variety of health complications [4,6]. However, less is known about differences in research participation comparing rural and urban populations or lower and higher socioeconomic groups [7,8]. Including representation from typically underrepresented groups in research will help scientists, healthcare providers, and policymakers better understand the specific needs of each group and, in turn, more appropriately inform treatments, recommendations and public health programming to be more inclusive [9].

The multi-site Pregnancy 24/7 cohort study (clinicaltrials.gov [NCT04749849]) aims to evaluate which 24-h movement behavior patterns (i.e., composition of physical activity, sedentary behavior, and sleep) lead to the healthiest pregnancies [10]. The West Virginia site of Pregnancy 24/7 used a recruitment method targeting potentially eligible pregnant women in their first trimester attending prenatal care visits at West Virginia University (WVU) Medicine-affiliated clinics in northcentral West Virginia and southwestern Pennsylvania. Women were sent information about the study through MyChart, a patient-facing portal linked to their electronic medical record.

To improve our understanding of clinical research participation in an observational pregnant cohort study from different underrepresented groups (i.e., rural and low socioeconomic status) in West Virginia, we conducted an analysis of the MyChart method recruitment data to understand proportions of screening and enrollment overall and among underrepresented groups. We hypothesized that proportions of participation would be lower in the participants who were rural or had public insurance compared to their better-represented counterparts (urban and private insurance).

## 2 Methods

### 2.1 Pregnancy 24/7 cohort study

Data for this study was from the multisite Pregnancy 24/7 cohort study (Iowa City, IA, Morgantown, WV, Pittsburgh, PA) which aims to understand how activity and sleeping patterns are associated with adverse pregnancy outcomes. The study enrolled participants from February 2021–May 2024. All follow-up visits were completed by October 2024. Specific details about Pregnancy 24/7 can be found on clinicaltrials.gov (NCT04749849) and in the published protocol [10]. Pregnant women were enrolled less than 23 weeks gestation and completed three study visits, one each trimester. Each visit participants wore two activity monitors and completed questionnaires. Though the study was designed to conduct the first visit in person and the second two remotely, fully remote participation was possible if needed. All study procedures in Pregnancy 24/7 were approved by a single institutional review board (202002630) at the University of Iowa (primary study site) with reliance from additional study sites, including WVU.

### 2.2 MyChart recruitment sub-study

The WVU site of Pregnancy 24/7 used MyChart to send recruitment messages. This method allows for quantification of individuals who were sent the recruitment information (the denominator), completed an initial screening questionnaire, and enrolled in the study. Therefore, the study sample included all individuals sent a MyChart recruitment message between October 2022–April 2023. MyChart (patient-facing platform linked to Epic electronic medical records) messages were sent to participants by an Epic medical record software team member. Participants received a notification through the app and email stating they received a message on MyChart. This method contacted people who attended a first trimester prenatal appointment in the previous week at a WVU Medicine-affiliated clinic and met the study's eligibility criteria based upon medical record review [10]. Individual obstetrics and gynecology providers permitted the research team to view their prenatal appointment schedules. The current analysis of the recruitment yield data was approved by the WVU Institutional Review Board (2205579603).

### 2.3 Measurements

We measured three proportions of participation. Screeners were those who completed an eligibility screening form. Consenters were individuals who enrolled in the study. Lastly, the proportion of Screen-to-consenters was the study enrollment proportion (i.e., consent) after screening. In other words, the Screen-to-consenters were eligible participants who filled out the screening form and then committed to participating in the study.

We defined underrepresented groups as those living in rural areas and those with low socioeconomic status [2,11]. Rurality was determined by first finding participants’ census-tract data (https://geomap.ffiec.gov/ffiecgeomap/) and entering this information into the 2010 Rural-Urban Commuting Area (RUCA) Codes database [12]. We used RUCA codes to categorize rurality as metropolitan (1–3), micropolitan (4–6), or small-town areas (7–10) [12]. We chose a three-group model to provide a more granular measure of rurality, as greater rurality may pose different barriers, such as longer commutes, to participating in clinical research. Insurance status was used as a proxy for socioeconomic status. Individuals were stratified by having private health insurance or public health insurance, i.e., Medicaid/Medicare. All remaining variables were abstracted from electronic medical records.

### 2.4 Data analysis

Analyses were conducted in R, version 4.1.1 (R Foundation for Statistical Computing, Vienna, Austria). Response proportions were calculated overall and per group for the Screeners, those who consented after screening (Screen-to-consenters), and Consenters out of all potential participants receiving the recruitment message. Logistic regression was used to determine differences in response proportions of underrepresented groups (more rural or receiving Medicaid/Medicare) compared to their typically better-represented counterparts. We did not adjust for covariates to understand the raw proportions of rurality and insurance status on research participation. Significant p-values were <0.5. Missing data was handled by multiple imputation using the mice package, version 3.15.0, with 5 imputed datasets.

## 3 Results

### 3.1 Response proportions

Of the 1024 people sent a recruiting message via MyChart, 7.5 % (n = 77) screened for Pregnancy 24/7, 89.6 % (n = 69) of whom were eligible to participate. Of the eligible screeners, 42 % (n = 29) consented to participate (i.e., Screen-to-consenters). The total yield of Consenters, those enrolling in the study after being sent the MyChart recruitment message, was 2.8 % (n = 29). A summary of participant characteristics can be found in Table 1.

**Table 1:** Summary of participant characteristics.

	Overall (N = 1024)
**Age (years)**^a^	27.7 (5.17)
**Rurality**	
Urban	520 (50.8 %)
Micropolitan Rural	238 (23.2 %)
Small-town Rural	194 (18.9 %)
Missing	72 (7.0 %)
**Race/Ethnicity**	
White/Caucasian	954 (93.2 %)
Underrepresented Racial Minority^b^	67 (6.5 %)
Missing/Refused to Answer	3 (0.3 %)
**Insurance Provider**	
Private	530 (51.8 %)
Medicaid/Medicare	480 (46.9 %)
Missing	14 (1.4 %)
**Marital Status**	
Married	579 (56.5 %)
Single	366 (35.7 %)
Significant Other	49 (4.8 %)
Separated, Divorced, or Widowed	25 (2.4 %)
Missing	5 (0.5 %)
**Prior Preterm Births**	
No	937 (91.5 %)
Yes	87 (8.5 %)
**Prior Nonviable Pregnancies**^c^	
No	709 (69.2 %)
Yes	300 (29.3 %)
**Prior Induced Abortions**	
No	983 (96.0 %)
Yes	26 (2.5 %)
**Living Children**	
None	444 (43.4 %)
One	338 (33.0 %)
Two or More	241 (23.5 %)

a Age expressed as mean (SD).

b Underrepresented racial minorities are Black/African American = 24, ‘Other’ = 17, Asian = 16, Hispanic/Latina = 8, Native American or other Pacific Islander = 2.

c Nonviable pregnancies include total spontaneous abortions and ectopic pregnancies.

**Fig. 1:**
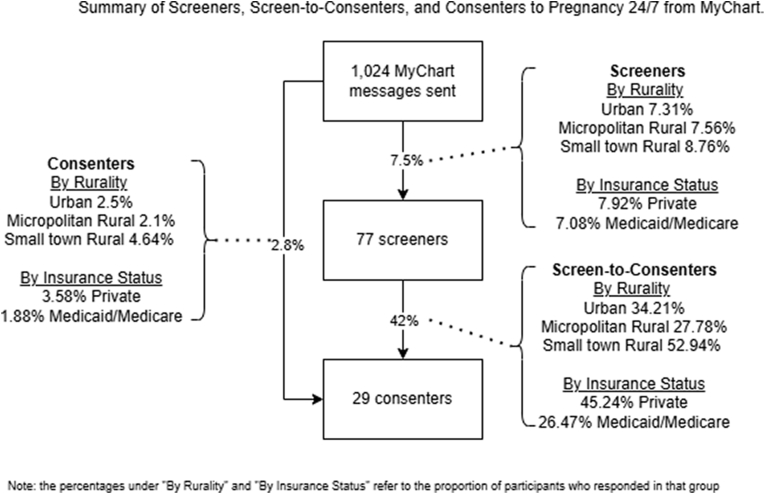
Summary of screeners, screen-to-consenters, and consenters to pregnancy 24/7 from MyChart.

Proportions of Screeners, Screen-to-consenters, and Consenters were not statistically different for underrepresented groups compared to their counterparts who are typically better represented in research (Table 2). Although not statistically different, those living in small-town rural areas had the highest relative proportions of Screeners, Screen-to-consenters, and Consenters to participate in Pregnancy 24/7. Proportions were approximately twice as high for the Screeners and Screen-to-consenters in small-town rural areas compared to urban areas. Regarding those with Medicaid/Medicare, though screening proportions were only slightly lower, only about half consented compared to those with private insurance. A summary of findings can be found in Fig. 1.

**Table 2:** Response proportions by population.

Population	Screeners (%)	Screeners OR (95 % CI)	Screen-to-Consenters (%)	Screen-to-Consenters OR (95 % CI)	Consenters (%)	Consenters OR (95 % CI)
**Rurality**						
Urban (n = 557)	7.31	Reference	34.21	Reference	2.50	Reference
Micropolitan Rural (n = 257)	7.56	1.04 (0.57–1.83)	27.78	0.74 (0.2–2.45)	2.10	0.84 (0.27–2.25)
Small-town Rural (n = 210)	8.76	1.22 (0.66–2.18)	52.94	2.16 (0.68–7.11)	4.64	1.90 (0.77–4.47)
**Insurance**						
Private (n = 538)	7.92	Reference	45.24	Reference	3.58	Reference
Medicaid/Medicare (n = 486)	7.08	0.89 (0.55–1.41)	26.47	0.44 (0.16–1.13)	1.88	0.51 (0.22–1.12)

Abbreviation: OR, odds ratios; CI, confidence interval; all analyses were run separately and unadjusted.

## 4 Discussion

The recruitment method in our study yielded 2.8 % enrollment which is similar to other studies that used health-care affiliated apps for recruitment [13,14]. Though there were no statistical differences in screening or consenting proportions when comparing underrepresented populations (i.e., individuals living in rural areas and those receiving Medicaid/Medicare) to their counterparts who are typically better represented in clinical research, consenting was about twice as likely in small-town rural individuals and half as likely among individuals with Medicaid/Medicare. This finding may be due to shared characteristics, such as younger age, and values of people who actively use and check their messages on MyChart [15].

Contrary to our hypothesis, there were similar participation proportions among rural and urban participation. The nature of our recruitment and data collection methods may have helped overcome barriers of those living in small-town rural areas [16,17]. First, regarding screening, participants did not need to leave their homes to be exposed to our recruitment advertisement. As for consenting, our participants may have been more likely to enroll due to our accommodating data collection methods, which were primarily remote, with a fully remote option offered, if needed. Also, the topic of our study may have encouraged rural participants to consent. Pregnancy 24/7 is a low-risk research study with a lower participant burden protocol (i.e., completing surveys, wearing activity monitors, and collecting anthropometric measurements). This may have allowed rural participants to overcome the barriers of mistrust and fear [16]. Lastly, as a pregnancy-related study, participants may have seen great value in participation. Previous research in rural populations reported that “benefits to family” was a strong motivator to participate in research [17]. Therefore, participation in this research may have been particularly appealing.

Participation proportions were similar for those with Medicaid/Medicare or Private insurance, however, consenting participants with Medicaid/Medicare was not as successful as those living in a rural setting. Screening proportions were similar compared to those receiving Private insurance which may be explained by exposure to the recruitment advertisement. However, these participants may have been hesitant to commit to participating in the study due to time constraints, competing responsibilities (e.g., work, childcare), low health literacy, or a mistrust in the research [18].

Our study has several strengths including the ability to define the denominator of potential participants who were sent our recruitment message and studying a special population of West Virginia and Appalachia with considerable geographic and socioeconomic diversity in typically underrepresented populations. However, our results may not be generalizable to research that does not share characteristics of our study, i.e., not pregnancy-related, observational, or does not offer remote participation options. We were also unable to conduct a meaningful analysis stratified by race due to the low, prevalence of non-White individuals (∼6.5 %) who were sent a recruitment message, which reflects West Virginia's demographic distribution.

Future research can expand upon our study in several ways. First, studies with recruitment strategies including different populations that include underrepresented racial minorities should evaluate if they are responsive to recruitment via MyChart. Also, given that overall participation was low (less than 3 % of those contacted enrolled), qualitative research such as interviews or focus groups to better understand barriers to responding to recruitment messages via healthcare-affiliated communication apps, overall and within underrepresented groups, will be critical to inform ways to improve representative recruitment in future studies.

## 5 Conclusions

Our findings suggest that MyChart may be a useful method for recruiting rural individuals into clinical research. Lower proportions of recruitment among individuals with Medicaid/Medicare insurance suggests other strategies are needed to improve research participation proportions in this group.

## CRediT authorship contribution statement

**Katrina L. Wilhite:** Writing – original draft, Methodology, Formal analysis, Data curation, Conceptualization. **Elly Marshall:** Writing – review & editing, Methodology. **Carly Williamson Rogers:** Writing – review & editing, Data curation. **Kara M. Whitaker:** Writing – review & editing, Methodology. **Lindsay Morris-Neuberger:** Writing – review & editing, Methodology. **Bethany Barone Gibbs:** Writing – review & editing, Supervision, Methodology, Formal analysis, Conceptualization.

## Funding sources

This research did not receive any specific grant from funding agencies in the public, commercial, or not-for-profit sectors.

## Declaration of competing interest

The authors declare that they have no known competing financial interests or personal relationships that could have appeared to influence the work reported in this paper.
