# Supplementary material for: Using healthcare-affiliated apps to recruit typically underrepresented populations in a pregnancy cohort study in West Virginia
Source: Contemp Clin Trials Commun. 2025 Aug 7;47:101535. doi: 10.1016/j.conctc.2025.101535 (PMC12356380; doi:10.1016/j.conctc.2025.101535)
Supplement: Multimedia component 1 [file mmc1.docx]

Join the Pregnancy 24/7 Research Study

Researchers at West Virginia University Medicine need your help to study how movement patterns and sleep are related to health during pregnancy. You may be eligible to join if you are less than 13 weeks pregnant. At three study visits, you will be asked to answer questionnaires and wear two movement monitors for 7 days. If preferred, visits can be completed virtually. We will also ask for your permission to gather pregnancy-related information from your medical record after you give birth. You can receive up to $150 if you complete all study visits.

To see if you are eligible, complete the screening form: {link to screening form}

To find out more information, please visit our website: {link to website}

If you have questions, contact us at (XXX) XXX-XXXX or [*preg.......@hsc.wvu.edu.*](mailto:preg247@hsc.wvu.edu)
